# Development of a Magnetic Molecularly Imprinted Microsphere-Based Signal Amplified Semi-Homogeneous Method for Multidetection of Five Progestins in Milk

**DOI:** 10.3390/foods12152818

**Published:** 2023-07-25

**Authors:** Yan Su, Gelin Liu, Haozhe Hou, Yaojia Peng, Jianping Wang

**Affiliations:** College of Veterinary Medicine, Hebei Agricultural University, Baoding 071000, China; sy20023995@163.com (Y.S.); lglssg2023@163.com (G.L.); 19831375921@163.com (H.H.); 15176963010@163.com (Y.P.)

**Keywords:** progesterone, magnetic molecularly imprinted microsphere, signal amplification system, semi-homogeneous method, progestins, milk

## Abstract

The residues of progestins in milk are significant risk factors for teenage acne and may cause hormone-dependent cancers in consumers, so the determination of these residues in milk is very important. However, an immunoassay or immunoassay-like method capable of determining multiple progestins in milk has not been reported so far. The present study, for the first time, synthesized a type of magnetic molecularly imprinted microsphere that was capable of simultaneously recognizing five progestins. At the same time, an enzyme labeled conjugate was synthesized by coupling progesterone 3-(o-carboxymethyl)oxime with streptavidinated horseradish peroxidase. The above two reagents were used to develop a semi-homogeneous method for the simultaneous detection of the residues of the five progestins in milk. During the experiments, biotinylated horseradish peroxidase was used to amplify the signal, so the sensitivity to the five drugs (limits of detection 0.04–0.1 pg/mL) was increased 44–75-fold. In addition, the magnetic molecularly imprinted microsphere could be regenerated four times by using simple elution. Through general comparison of its detection spectrum, sensitivity, simplicity, and reusability, the present method exhibited better performance than the previous immunoassays for the detection of progestins, and so it could be used as a routine tool for the screening of progestins residues in milk.

## 1. Introduction

**Figure 1:**
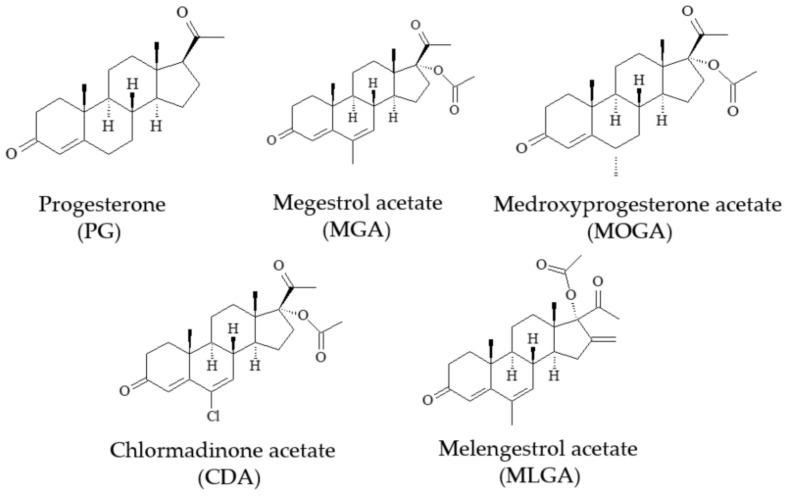
Molecules of five commonly used progestins.

Progesterone (PG, Figure 1) is a type of natural progestin that regulates the activity of sexual organs and maintains the female reproduction system. In addition, other synthetic progestins (medroxyprogesterone acetate (MOGA), megestrol acetate (MGA), chlormadinone acetate (CDA), and melengestrol acetate (MLGA) (Figure 1)) have also been used to promote regular menstrual cycles and control the symptoms of menopause. For dairy cows, these progestins can be used as growth promoters, but their residues in milk represent huge risks to consumers [1,2]. For example, a long period of consuming progestin-containing milk may interfere with the growth of teenage acne and cause breast, ovarian, and corpus uteri cancers in consumers. As a result, the Ministry of Agriculture of China lists melengestrol acetate as a banned drug for food-producing animals [3] and sets a maximum residue limit of 1 μg/kg for flurogestone acetate in milk [4]. For the protection of consumer health, the inspection of the residues of these progestins in milk is very important.

Up to now, many methods including instrumental techniques [5] and immunoassays [6] have been reported to detect progestins in various food samples. The commonly used instrumental methods, e.g., liquid chromatography tandem mass spectrometry, are qualitative and quantitative determination tools but expensive equipment, a special laboratory, and a professional operator are required, so these methods are not suitable for the analysis of a large number of samples. In contrast, immunoassays based on 96-well microplates are simple, rapid, and capable of analyzing dozens of samples in one assay, and the cost is low, so they are a commonly used screening tool for a large number of samples. So far, various immunoassays, including the enzyme-linked immunosorbent assay [7,8], fluorescence immunoassay [9,10,11], chemiluminescence immunoassay [12], immunochromatographic strip [13,14,15,16], and biosensors [17,18,19], have been reported to detect progestins. However, most of the above immunoassays can only determine PG, only one immunoassay can determine MLGA, and immunoassays capable of detecting other progestins have not been reported. This means that the previously reported immunoassays for progestins can only detect one drug; in other words, an immunoassay capable of determining multiple progestins in foods of animal origin has not yet been reported.

All of the immunoassays mentioned above utilize polyclonal antibodies or monoclonal antibodies as the recognition reagents. It is well known that the production of an antibody requires many laboratory animals, the process is time-consuming, and the cost is high. So, finding other specific recognition reagents is necessary. Molecularly imprinted polymer (MIP), also called “plastic antibody”, is a type of specific recognition reagent that is synthesized with a specific molecule as the template. The formed 3D cavities in the MIP show specific recognition of the template and its structurally similar molecules. For an MIP, the synthesis process only needs several days, the procedure is simple, the cost is low, and the product is recyclable. Therefore, many researchers use MIP as the recognition reagent to develop various analysis methods for the determination of different analytes [20,21,22], such as the pseudo enzyme-linked immunosorbent assay [23], pseudo chemiluminescence immunoassay [24], pseudo fluorescence immunoassay [25], and pseudo immunochromatographic strip [26]. The results of these reports show that MIP-based pseudo immunoassays are better than or comparable to the related immunoassays. So, MIP is a promising recognition reagent.

Up to now, only several papers have reported the synthesis of MIP for progestins (PG), and the synthesized MIP films are used to prepare several sensors for the detection of PG [27,28,29,30]. However, the preparation processes of these sensors are complicated, and the prepared sensors can only detect PG in water or urine samples. To the best of our knowledge, however, a MIP material capable of recognizing other progestins has not been reported so far, and a MIP-based pseudo immunoassay capable of detecting PG or other progestins has not been reported either.

**Figure 2:**
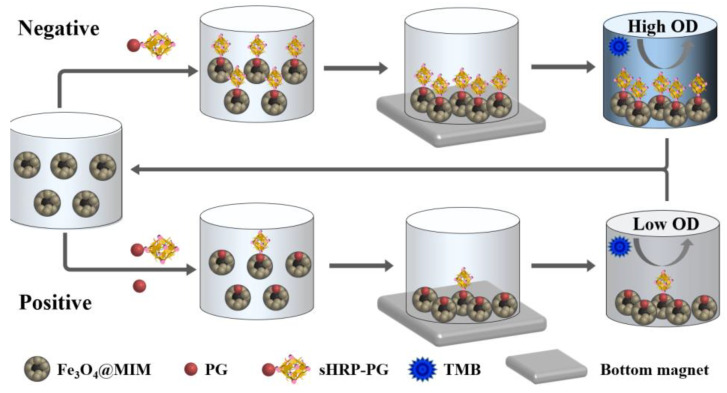
Operation route of the semi-homogeneous method.

In the present study, a type of magnetic molecularly imprinted microsphere capable of simultaneously recognizing five progestins was first synthesized with PG as the template, and this magnetic composite was used to develop a semi-homogeneous method on a 96-well microplate with the help of a 96-well bottom magnet. As shown in Figure 2, the magnetic composite, the enzyme labeled conjugate, and the progestins were directly added into microplate wells for competition; this meant that the microplate coating, blocking, and the related washing steps were avoided, thus, reducing operation steps and shortening assay time. After that, the solutions were discarded with the assistance of a bottom magnet. Then, the magnetic complexes were washed, and the optical signal was induced. Finally, the magnetic composite was regenerated for the next use. In this method, biotinylated horseradish peroxidase was used to amplify the signal, so the sensitivity was increased up to 75 times with the limit of detection down to pg/mL level. General comparison: the performances of this method were better than the previously reported immunoassays and MIP-based methods for progestins, so it should be a promising tool for inspection of the five progestins in large number of milk samples.

## 2. Materials and Methods

### 2.1. Chemicals

Progesterone (PG, purity 98%), medroxyprogesterone acetate (MOGA, purity 98%), megestrol acetate (MGA, purity 96%), divinylbenzene (DVB, purity 80%), and Fe_3_O_4_ were purchased from Aladdin Biochemical Technology Co., Ltd. (Shanghai, China). Chlormadinone acetate (CDA, purity 98%) and melengestrol acetate (MLGA, purity 98%) were purchased from Shanghai Makclin Biochemical Co., Ltd. (Shanghai, China). Biotinylated horseradish peroxidase, streptavidinated horseradish peroxidase, progesterone 3-(o-carboxymethyl)oxime (purity 95%), tetramethylbenzidine (purity 98%), and sulfuric acid (purity 98.3%) were purchased from Shanghai Yuanye Biological Technology Co., Ltd. (Shanghai, China). Methacrylic acid (MA, purity 99.5%) and 2,2-azobis (isobutyronitrile) (AIBN, purity 95%) were purchased from Tianjin Kemiou Chemical Reagent Co., Ltd. (Tianjin, China). Additionally, 1-ethyl-3-(3-dimethylaminopropyl)-carbodiimidehydrochlorde (EDC, purity 97%) and N-hydroxysuccinimide (NHS, purity 98%) were purchased from Sigma-Aldrich (St. Louis, MO, USA).

### 2.2. Synthesis of Fe_3_O_4_@MIM

In this study, PG was used as the template molecule to synthesize the magnetic molecularly imprinted microsphere (Fe_3_O_4_@MIM), which was carried out according to the principle of our previous report [31]. During the experiments, 0.1 mmol PG and 0.4 mmol function monomer MA were dissolved in 20 mL acetonitrile, and this solution was put into a tube to be pre-polymerized for 3 h at room temperature. Then, 4 mmol DVB, 40 mg AIBN, and 50 mg Fe_3_O_4_ were added, and the mixture was blown with nitrogen stream for 30 min. After that, the tube was capped and put into a 60 °C water bath to be stirred for 24 h. Then, the sediments were separated with the assistance of a magnet, and the solution was discarded. The products left were washed with 10 mL methanol/acetic acid (9:1, *v*/*v*) for 10 cycles. The obtained Fe_3_O_4_@MIM products were washed with water and dried for use. At the same time, a non-imprinted composite (Fe_3_O_4_@NIM) was also synthesized, but without the addition of the template PG.

### 2.3. Synthesis of Enzyme Labeled Conjugate

The enzyme labeled conjugate (sHRP-PG) was prepared by coupling the hapten progesterone 3-(o-carboxymethyl)oxime with streptavidinated horseradish peroxidase (sHRP) by using active ester method. During the experiments, the hapten (40 μmol), EDC (20 μmol), and NHS (20 μmol) were dissolved in 2 mL dimethylformamide, and this solution was stirred at 4 °C for 6 h. At the same time, 1 μmol sHRP was dissolved in 1 mL PBS, and this solution was added into the above solution to be stirred at 4 °C overnight. Finally, the solution was transferred into a dialysis bag for dialyzing against PBS for 72 h to obtain the sHRP-PG.

### 2.4. Development of Semi-Homogeneous Method

In this study, the semi-homogeneous method was performed as the route shown in Figure 2. First, 2 mg Fe_3_O_4_@MIM was suspended in 5 mL distilled water to obtain a homogeneous suspension. Second, 50 μL suspension (containing 20 μg Fe_3_O_4_@MIM), 50 μL analyte solution, 50 μL sHRP-PG, and 50 μL biotinylated horseradish peroxidase (bHRP) were directly added into the microplate wells for semi-homogeneous competition (37 °C 10 min). Third, the supernatants in the wells were discarded under the assistance of a 96-well bottom magnet, and the Fe_3_O_4_@MIM particles left were rinsed with water for 3 cycles. Fourth, 100 μL tetramethylbenzidine was added to be incubated at 37 °C for 10 min, and 50 μL 2 M sulfuric acid was added to terminate the reaction. Fifth, the plate was put into an ELISA reader, and the optical densities (OD_450 nm_) of these wells were recorded. Finally, the Fe_3_O_4_@MIM particles left in the wells were collected and eluted with 20 mL methanol/acetic acid (9:1, *v*/*v*) for the next test. The concentrations showing 50% of inhibition (IC_50_) and the concentrations showing 10% of inhibition (IC_10_, limit of detection) for the 5 progestins were determined, respectively, and the two parameters without the addition of bHRP were also calculated.

### 2.5. Sample Preparation and Method Application

The sample pretreatment was performed according to a method from previous report [11]. An appropriate volume of milk sample was diluted 10-fold with water to be assayed directly without additional pretreatment. For evaluation of the method, the 5 progestins were spiked into the blank milk samples obtained from several controlled dairy farms at levels of 0.1, 1.0, and 10 ng/mL, and the spiked samples were diluted and assayed as described in the above procedure, respectively. For evaluation of the method application, 60 commercial milk samples purchased from several local supermarkets were analyzed.

## 3. Results and Discussions

### 3.1. Fe_3_O_4_@MIM

**Figure 3:**
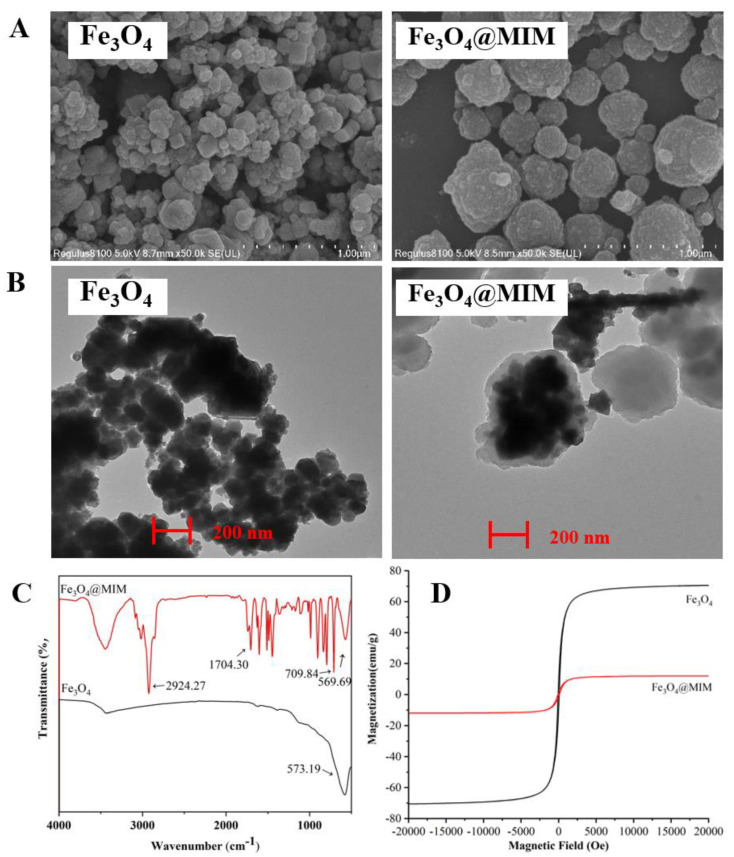
Characterization results forFe_3_O_4_@MIM by using (**A**) SEM, (**B**) TEM (red scale, 200 nm), (**C**) FTIR, and (**D**) VSM.

In the present study, a type of magnetic molecularly imprinted polymer with PG as the template molecule was first synthesized. The characterization result from the scanning electron microscope (SEM) showed that the Fe_3_O_4_@MIM products were generally sphere particles (Figure 3A). Their mean diameter was 296 nm, much larger than the Fe_3_O_4_ particles (125 nm), which meant that the polymer was polymerized on Fe_3_O_4_. This result was consistent with the characterization result from the transmission electron microscope (TEM), which showed that the Fe_3_O_4_@MIM was a type of core–shell structured material (Figure 3B).

The characterization result from Fourier transform infrared (FTIR) showed that Fe_3_O_4_@MIM was a kind of composite, because it contained the characteristic peaks of the functional monomer (C-H 2924.27 cm^−1^, C=O 1704.30 cm^−1^, C=C 709.84 cm^−1^) and the Fe_3_O_4_ (569.69 cm^−1^) (Figure 3C). The hysteresis loops from vibrating sample magnetometry (VSM) showed that the magnetic properties of Fe_3_O_4_@MIM and Fe_3_O_4_ were very similar (Figure 3D), which meant that Fe_3_O_4_@MIM was a magnetic material. The above-mentioned results proved that the designed magnetic composite Fe_3_O_4_@MIM was synthesized.

### 3.2. Selective Absorption Ability of Fe_3_O_4_@MIM

**Figure 4:**
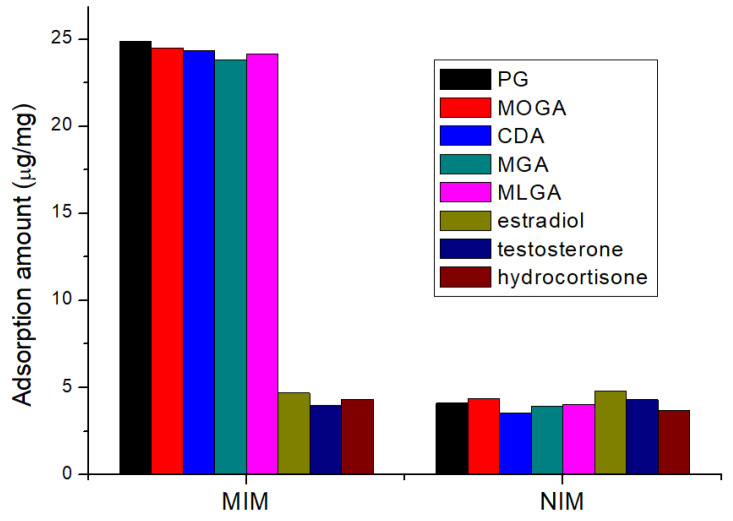
Mean absorption amounts of Fe_3_O_4_@MIM and Fe_3_O_4_@NIM for progestins and other analytes (*n* = 5).

A type of MIP usually shows the specific recognition for the template and its structurally similar molecules. For evaluation of the selective absorption ability, the adsorption amounts of the Fe_3_O_4_@MIM and the Fe_3_O_4_@NIM for the five progestins and three other steroid hormones (estradiol, testosterone, hydrocortisone) were determined. When using the Fe_3_O_4_@MIM, the adsorption amounts for the five progestins (23.8–24.9 μg/mg) were much higher than that for other steroid hormones (3.96–4.48 μg/mg) (Figure 4). When using the Fe_3_O_4_@NIM, the adsorption amounts for all of these analytes were comparably low (3.54–4.8 μg/mg). These results proved that the present Fe_3_O_4_@MIM only recognized progestins. The previously reported PG-based MIP films only recognized PG, and the present Fe_3_O_4_@MIM could recognize five progestins, so its broad recognition ability was better than those of MIP films [27,28,29,30].

### 3.3. Characterization of Enzyme Labeled Conjugate

**Figure 5:**
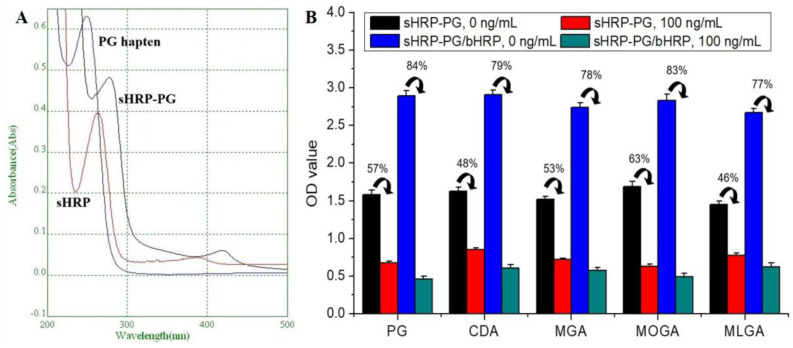
(**A**) UV diagram of sHRP−PG, and (**B**) OD values when testing the five progestins when using sHRP−PG/bHRP system and sHRP−PG (Fe_3_O_4_@MIM, 50 μg/well; sHRP−PG/bHRP and sHRP−PG, 10 μg/mL (determined as pure HRP); incubation 30 min).

sHRP-PG, sHRP, and the hapten progesterone 3-(o-carboxymethyl)oxime were all characterized with a UV spectrophotometer to identify the conjugation, because a conjugate should contain the characters of the hapten and the carrier. As can be seen from the scanning diagrams shown in Figure 5A, the sHRP-PG contained the characteristic peaks of PG hapten and sHRP, revealing the sHRP and the PG hapten were linked and the sHRP-PG was obtained.

### 3.4. Evaluation of the Two Signal Systems

To verify if the use of bHRP could amplify the signal, the following experiments were conducted. The Fe_3_O_4_@MIM particles were added into the microplate wells, and then the five progestins were mixed with sHRP-PG and sHRP-PG/bHRP to perform a semi-homogeneous competition, respectively. As shown in Figure 5B, when the analyte concentration was zero, the use of sHRP-PG/bHRP system and single sHRP-PG could all induce high optical signals, and this meant that the conjugate sHRP-PG could bind with the Fe_3_O_4_@MIM. However, the optical density values from the sHRP-PG/bHRP system were much higher than that from the single sHRP-PG. This was because the sHRP-PG could bind four bHRP molecules, and the five HRP molecules in sHRP-PG/bHRP system catalyzed the high signal. When the analyte concentration was 100 ng/mL, the optical density values all decreased, no matter which signal system was used (Figure 5B). This proved that the Fe_3_O_4_@MIM and the sHRP-PG could be used to develop a competitive detection method for progestins. In contrast, the use of sHRP-PG/bHRP achieved much higher inhibition ratios for the five drugs (1-B/B_0_, 77–84%, where B is the OD value at 100 ng/mL, and B_0_ is the OD value at zero concentration) than the use of single sHRP-PG (46–63%). This proved that the use of sHRP-PG/bHRP system could increase the method sensitivity, which was consistent with our original expectation.

### 3.5. Optimization of Operation Parameters

**Figure 6:**
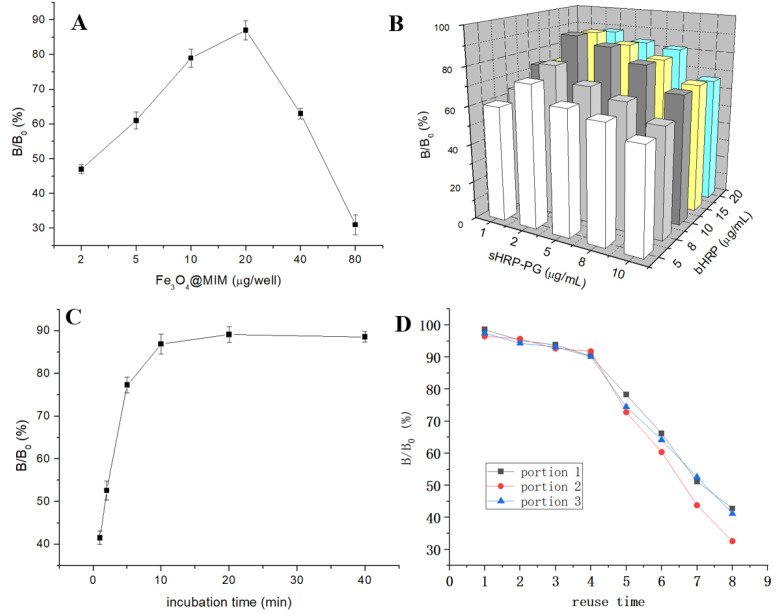
B/B_0_ values of PG (10 ng/mL) (**A**) at different concentrations of Fe_3_O_4_@MIM (bHRP 10 μg/mL, sHRP-PG 10 μg/mL, incubation 30 min), (**B**) at different concentrations of sHRP-PG/bHRP (incubation 30 min), (**C**) at different incubation times (Fe_3_O_4_@MIM 20 μg/well; bHRP 10 μg/mL, sHRP-PG 2 μg/mL), and (**D**) after different times of reuse.

During the experiments, PG was used as the representative drug to optimize several parameters. First, the amounts of Fe_3_O_4_@MIM (2–50 μg/well) were optimized. As shown in Figure 6A, the inhibition ratio of PG reached the highest when using 20 μg/well of Fe_3_O_4_@MIM, so it was selected as the optimal condition. Second, the concentrations of sHRP-PG and bHPR were optimized. As shown in Figure 6B, the inhibition ratio of PG reached the highest when using 2 μg/mL of sHRP-PG and 10 μg/mL of bHPR, so the two enzyme concentrations were selected as the optimal conditions. Third, the mixture was incubated for different times, and the result showed that the inhibition ratio of PG reached equilibrium when the semi-homogenous competition was performed for 10 min (Figure 6C), so it was selected as the optimal incubation time. Under the above optimized conditions, the Fe_3_O_4_@MIM particles (2 mg, three portions) were successively regenerated eight times to evaluate the recycle performance. As shown in Figure 6D, the inhibition ratios were higher than 90% when they were regenerated 1–4 times, but lower than 80% after being reused 5 times. This was possibly because the imprinted cavities in the composite were occupied by analyte molecules that could not be fully removed, or some of the composite particles were missing during the repeated uses. So, the composite was reused four times in the subsequent experiments.

### 3.6. Method Performances

**Figure 7:**
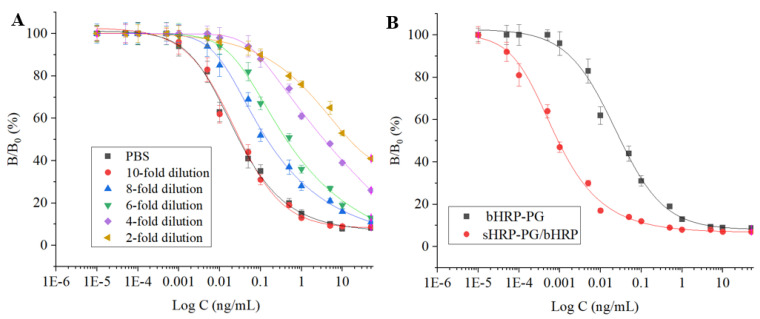
The competitive inhibition curves of PG fortified blank milk (**A**) at different dilutions by using sHRP-PG/bHRP and (**B**) at 10-fold dilution when using the two signal systems (0.00001–50 ng/mL).

For a rapid screening method, the simple sample extraction method was very important. For evaluation of the matrix effect, PG was fortified into the blank milk sample and diluted for different folds to be assayed directly. As shown in Figure 7A, the competitive curve after 10-fold dilution was identical with that of PG in PBS, which meant that the sample impurities could be omitted after 10-fold dilution. Then, the five drugs at different concentrations were prepared with the 10-fold diluted milk sample to be tested. Results showed that the IC_50_ of the five progestins were in the range of 0.8–1.8 pg/mL, and the limits of detection (IC_10_) were in the range of 0.04–0.1 pg/mL (Table 1). During the experiments, the two parameters for the five progestins when using the single sHRP-PG were also determined (IC_50_ of 22–52 pg/mL, limits of detection of 3.0–6.0 pg/mL, Table 1). This meant that the limits of detection (IC_10_) for the five analytes were improved 44–75-fold when using the signal amplification system (Table 1) consistent with the original expectation. The competitive curves of PG when using sHRP-PG/bHRP and single sHRP–PG, shown in Figure 7B, proved the sensitivity improvement effect. Including within the 10-fold sample dilution, the limits of detection of the present method for determination of the five progestins in milk were in the range of 0.4–1.0 pg/mL.

### 3.7. Method Application

For the evaluation of its application, the five progestins were spiked into the blank milk sample for analysis. As shown in Table 2, the intra-day recoveries of the five progestins from the standards fortified blank milk samples (six repetitions in one day) were in a range of 65.4–88.2%, and the inter-day recoveries (duplicate analysis on six successive days) were in a range of 69.4–87.2%. Finally, the 60 commercial milk samples were diluted and assayed as in the above procedures. Results showed that only two out of the 60 samples were determined as positive (sample A 4.5 ng/mL, sample B 1.9 ng/mL; calculated as PG), but the specific drug could not be confirmed. This indicated that the positive result determined by the semi-homogeneous method should be validated with an instrumental method to identify the specific drug, and such instrumental method capable of determining the five progestins in milk remain to be studied. Still, the Fe_3_O_4_@MIM-based semi-homogeneous method developed in this study could be utilized as a routine screening tool for the rapid detection of the five progestins in milk.

### 3.8. Comparison with Immunoassays and MIP-Based Methods for Progestins

The present study, for the first time, reported a Fe_3_O_4_@MIM-based semi-homogeneous method to determine the presence of five progestins in milk. In comparison with the present method, the main results of previous immunoassays and MIP-film-based sensors for the determination of progestins were collected in Table 3. First, the present method could determine five progestins, which was better than all of those methods. Second, the limit of detection of the present method was down to pg/mL level, which was better than most of those methods. Third, one assay of the present method lasted 20 min, which was shorter than most of those methods. Fourth, the conventional immunoassays could not be recycled whereas the present method could be regenerated four times. Though several previous biosensors are reusable, they require special instruments, which limits their wide applications. Due to their general comparison of detection spectrum, sensitivity, reusability, and assay time, the Fe_3_O_4_@MIM-based semi-homogeneous method exhibited better performances than those methods.

## 4. Conclusions

Progesterone and the synthetic progestins are usually used for dairy cows, but their residues in milk are dangerous to consumers. Therefore, the inspection of their residues in milk is necessary. However, the previously published immunoassays and molecularly imprinted polymer-based methods could only determine one drug. The present study synthesized, for the first time, a magnetic molecularly imprinted microsphere capable of simultaneously recognizing five progestins. Then, the magnetic composite was used to develop a semi-homogeneous method on a 96-well microplate under the assistance of 96-well bottom magnet. The operation of this method was simple and rapid, and the magnetic composites could be recycled 4 times. Due to the use of biotinylated horseradish peroxidase and streptavidinated horseradish peroxidase system, the method signal was highly enhanced, and the sensitivities were increased 44–75-fold with the limit of detection down to pg/mL level. The general method performances were better than the previous methods for detection of progestins. Therefore, the present method could be utilized as a routine tool for the rapid screening of the five progestins in a large number of milk samples. With the guidance of this study, more magnetic molecularly imprinted polymer-based semi-homogeneous methods for the detection of other substances may appear in the future, and the signal amplification strategy of the present method may inspire other researchers to find and create more signal amplification strategies.

## Figures and Tables

**Table 1:** Determination parameters for the five progestins.

Analyte	sHRP-PG		sHRP-PG/bHPR		IC_10_ Improvement Fold
	IC_50_ (pg/mL)	IC_10_ (pg/mL)	IC_50_ (pg/mL)	IC_10_ (pg/mL)	
PG	24	3	0.9	0.05	60
CDA	35	4	1.1	0.09	44
MGA	22	3	0.8	0.04	75
MOGA	47	5	1.5	0.08	63
MLGA	52	6	1.8	0.1	60

**Table 2:** Recoveries of the Fe_3_O_4_@MIM-based semi-homogeneous method for the five progestins.

Analyte	Added (ng/mL)	Intra-Day		Inter-Day	
		Recovery (%)	CV (%)	Recovery (%)	CV (%)
PG	0.1	65.4	7.3	69.4	9.3
	1.0	76.2	8.2	83.2	8.5
	10	79.3	6.4	80.4	10.6
CDA	0.1	70.6	8.2	75.3	9.7
	1.0	83.4	6.7	79.5	13.4
	10	81.0	6.1	71.3	10.6
MGA	0.1	67.4	8.8	74.3	11.8
	1.0	74.2	8.2	79.6	8.9
	10	79.3	8.4	85.0	7.3
MOGA	0.1	69.5	9.4	71.5	14.2
	1.0	82.5	7.3	83.2	10.3
	10	85.3	7.0	87.3	8.8
MLGA	0.1	72.5	8.2	75.3	11.2
	1.0	81.5	8.5	77.8	12.4
	10	88.2	8.4	70.6	10.7

**Table 3:** Results of previously reported immunoassays and MIP-based methods for progestins.

Recognition Reagent	Method	Analyte	Assay Time (from Addition of Sample)	LOD (ng/g)	Sample	Reusable?	Ref.
pAb	ELISA	PG	Overnight	0.0015	milk	No	[7]
pAb	ELISA	MLGA	Overnight	0.1	beef	No	[8]
pAb	chemiluminescence immunoassay	PG	90 min	0.06	human serum	No	[9]
mAb	fluorescence immunoassay	PG	13 min	5.0	human serum	No	[10]
mAb	fluorescence immunoassay	PG	12 min	0.065	milk	No	[11]
mAb	fluorescence immunoassay	PG	--	0.1	milk	No	[12]
mAb	surface plasmon resonance sensor	PG	5 min	3.56	milk	Yes	[17]
mAb	chemiluminescence sensor	PG	35 min	1.2	human serum	Yes	[18]
mAb	surface plasmon resonance sensor	PG	45 min	0.5	milk	Yes	[19]
MIP film	electrochemical sensor	PG	30 min	0.001	human urine	Yes	[27]
MIP film	surface plasmon resonance sensor	PG	16 min	3.1 × 10^−6^	water	Yes	[28]
MIP film	surface plasmon resonance sensor	PG	20 min	0.9 × 10^−9^	water	Yes	[29]
MIP film	optical sensor	PG	12 min	0.5	water	Yes	[30]
Fe_3_O_4_@MIM	semi-homogeneous method	5 drugs	20 min	0.0004–0.001	milk	Yes	This study

## Data Availability

The data presented in this study are available on request from the corresponding author.
